# The Care of the Poor

**Published:** 1895-01

**Authors:** 


### THE CARE OF THE POOR.

One of the most important questions relating to health economics in a great city like Buffalo is that of properly caring for its poor. It is entirely within the province of physicians and medical magazines to discuss this subject in all seriousness as one closely allied to the great problem of preventive medicine.

If the highest function of prevention is exercised, it must be done through channels that relate to the health of the poor. Disease can be prevented in a great measure by maintenance of nutrition, warmth and cleanliness. It is better to prevent sickness than to cure it. This aphorism holds good viewed from the most severe standpoint of money economics.

There is no surer way to breed disease than through the avenues of starvation and nakedness. Fuel, food and clothing are great sanitary allies and should be dealt out with a liberal hand as preventive measures. This applies equally to public and private charity.

Let it not be understood that we advocate recklessness in giving through either of these channels. On the contrary, it should be done with the greatest circumspection.

The individual pauper is a fellow-man suffering from social laws for which he is not responsible, and is, therefore, entitled to the kindest consideration through organized charity. The public pauper is the ward of the great body politic and his guardians should be compelled to protect him if they do not do it willingly.

Our argument is briefly this : that in times of financial stringency like the present, when the number of the poor is extraordinarily great, every effort should be put forth to care for them in an adequate manner with a view to the prevention of sickness and death. No city with the wealth of Buffalo can afford the reputation of an increased sickness or death-rate arising from neglect. Every avenue of charity should be drawn upon to the limit of its ability. Wealthy and well-to-do citizens should sustain the charity organization society with liberal hands. The present appeal of this society to such should not go unheeded.

Finally, the municipal authorities, executive and legislative, should put forth the utmost exertion and administer their functions with even a strained liberality, with a view to employing laboring men and women in order to prevent distress and thus sickness and death.

